# Effect of Work-Related Sedentary Time on Overall Health Profile in Active vs. Inactive Office Workers

**DOI:** 10.3389/fpubh.2018.00279

**Published:** 2018-10-01

**Authors:** Pauline M. Genin, Pascal Dessenne, Julien Finaud, Bruno Pereira, Frederic Dutheil, David Thivel, Martine Duclos

**Affiliations:** ^1^Clermont Auvergne University, EA 3533, Laboratory of the Metabolic Adaptations to Exercise under Physiological and Pathological Conditions (AME2P), Clermont-Ferrand, France; ^2^CRNH-Auvergne, Clermont-Ferrand, France; ^3^INRA, UMR 1019, Clermont-Ferrand, France; ^4^University Clermont 1, UFR Medicine, Clermont-Ferrand, France; ^5^Caisse Primaire d'Assurance Maladie, Clermont-Ferrand, France; ^6^Association Sportive Montferrandaise, Clermont-Ferrand, France; ^7^Clermont-Ferrand University Hospital, Biostatistics Unit (DRCI), Clermont-Ferrand, France; ^8^School of Exercise Science, Australian Catholic University, Sydney, NSW, Australia; ^9^Occupational Medicine, University Hospital CHU G. Montpied, Clermont-Ferrand, France; ^10^Department of Sport Medicine and Functional Explorations, Clermont-Ferrand University Hospital, G. Montpied Hospital, Clermont-Ferrand, France

**Keywords:** tertiary employees, physical activity, sedentariness, health, fitness

## Abstract

**Objective:** While public health strategies are developed to fight sedentary behaviors and promote physical activity, some professional activities, and especially tertiary ones, have been pointed out for their highly sedentary nature. Although workplace physical activity programs are increasingly proposed by companies to their employees in order to increase their physical activity levels, sitting and screen time remain extremely high. The main aim of this work was to compare health indicators between active and inactive tertiary employees with similar high levels of sedentariness. Secondly, we questioned the effects of a 5-month workplace physical activity program on overall health indicators among initially active and inactive tertiary employees.

**Methods:** Anthropometric measurements, body composition (bio-impedance), physical fitness (cardiorespiratory and musculoskeletal fitness) and health-related quality of life and perception of health status (self-reported questionnaires) were assessed among 193 active and inactive tertiary employees before (T0) and after a 5-month workplace physical activity intervention (T1), composed of 2 physical sessions per week.

**Results:** Significant improvements were found in performance of push-ups (*p* < 0.001), back muscle strength (*p* < 0.001) fat mass (*p* < 0.01) and waist circumference (*p* < 0.05) in active compared with inactive employees both at baseline and at the end of the program. Health perception (*p* < 0.001) was significantly different between groups at T0 but not at T1. However, no significant difference was observed for fat-free mass, BMI, workplace well-being and lower and upper limbs muscle strength. The variations between T0 and T1 demonstrate that, while all the studied parameters progressed positively during the 5-month program, health perception (*p* < 0.001), back muscle strength (*p* < 0.05) and BMI (tendency) showed a significantly higher progression in the inactive compared with the active group.

**Conclusion:** Health indicators might not be improved among active tertiary employees compared with inactive ones, which might be due to the high level of sedentariness characterizing their occupational task.Structured on-site physical activity programs can improve health in both initially active and inactive employees.

## Introduction

Over the last decades, sedentariness has become one of the largest public health concerns, recognized as one of the main causes of preventable premature mortality. Diaz et al.recently highlighted that both the total volume of sedentary time as well as its accrual in prolonged, uninterrupted bouts are associated with increased all-cause of mortality (1), which has been associated with poor health at all ages, independently of the level of physical activity (2). It is crucial to clarify that sedentariness is not the same as lack of physical activity, as people can reach the recommended levels of physical activity for their age, yet spend a large amount of their time engaging in sedentary activities (3, 4). Evidence suggests that the time dedicated to sedentary activities (leisure and work) has increased from 26 to 38 h per week between 1965 and 2009 in the United States and from 30 to 42 h between 1960 and 2005 in Great Britain, expressing alarming prospects for 2030 (5). According to the 2006 national nutrition and health survey (ENNS), 53% of adults aged 18 to 74 (59% of men and 48% of women) spend 3 h or more per day (working days and holidays) in front of a screen (television or computer) outside of working hours. Importantly, this proportion progresses with age in both men and women.

When it comes to work-related sedentary time particularly, it appears that the prevalence of sedentary professions increased by 20% in the United States between 1960 and 2008, with a concomitant decline of more “physically active professions” (5). In France, working adults have been shown to spend on average 9.96 h per day sitting on workdays (with at least 4.17 h/day seated at work) and 7.58 h/day sitting on non-workdays (6). Importantly, Saidj et al. reported clear associations between the sedentary time at work and the adoption of sedentary behaviors outside work.

Worldwide, public health policies underline the urgent need to create a suitable culture of regular physical activity (7), where employers are encouraged to play a key role in the promotion of health and well-being among adults of working age (8).

Workplaces represent today an ideal opportunity for new initiatives to promote physical activity. Workplaces could indeed reduce some of the barriers that have been identified to limit the engagement in physical activity, such as lack of time and proximity (9). Due to the difficulties in changing the habits of populations and to promote physical activity in usual settings (such as associations, gymnasiums, etc.), workplace-based programs might provide a great way to incite employees to increase their activity levels, especially due to the amount of time people spend at their workplace (10). There is clear evidence that increasing the employees' level of physical activity has beneficial effects on their health, concomitantly reducing health care costs (11) and the cost of different treatments for preventable diseases, and decreasing the number of sick leave due to diseases or injuries (12, 13). Some previous studies have indeed shown that interventions conducted to improve employees' health, may lead to reduced absenteeism and sick leave, while favoring increased productivity (14, 15).

Although some controlled and well-designed studies have found that workplace-based physical activity interventions can improve general health, physical activity levels (14, 16), weight status (17) and may have positive effects on eating behavior among employees (18); a recent systematic review identified some limitations of such interventions, due to large inter-individual heterogeneity (19). At the same time, a recent study conducted in 2017, underlined the beneficial effects of worksite physical activity programs proposed to tertiary employees on overall health (20). Although this pilot study was the first to our knowledge to enroll both experimented (active individuals, already regular users of their companies' physical facilities) and novice (inactive before the intervention) participants, further studies are now needed using larger sample sizes and more objective methods. Moreover, while the studies conducted so far mainly focused on increasing the level of physical activity of workers, tertiary employees remain highly sedentary due to the static nature of their work, which might have deleterious effects, independently of their physical activity levels. Some interesting studies effectively suggest that long periods of sedentary behaviors increase the risk of cardiovascular diseases, type 2 diabetes, some cancers and obesity, among other conditions, even in individuals reaching recommended levels of physical activity (2).

While it remains evident that workplace interventions have to be conducted to favor healthy active living among tertiary employees, the sedentary nature of such professional activities must be considered. The first aim of the present study was to compare overall health indicators among physically active and inactive tertiary employees, showing a high level of sedentariness. Secondly, we assessed the effects of a 5-month physical activity workplace intervention on overall health indicators between sedentary previously active and newly active tertiary employees.

## Methods

### Participants and design

A total of 193 office employees (tertiary workers; 83 females, 110 males; age: 44.2 ± 9.8 years; weight: 72.6 ± 14.7 kg; BMI: 24.5 ± 3.8 kg/m^2^) took part in this quasi-experimental study. Participants were approached and recruited through the manufacturer internal network, thanks to various informative announcements to the employees. After a medical inclusion to control for their ability to complete the whole study, anthropometric measurements and body composition were assessed, aerobic fitness, muscle capacities, well-being as well as quality of life and health perception were measured (T0). The participants followed then a 5-month on-site physical activity intervention and all these measures were replicated by the end of the intervention (T1). All the participants received information sheets and signed consent forms as requested by ethical regulations. To be included the participants had to: (i) be tertiary employees; (ii) show no contraindications to physical practices; (iii) be free of any medication that could interfere with the study outcomes. Employees who showed a regular participation in their worksite physical activity program for the last 2 years were classified as active while the others were classified as inactive. Their physical activity level was also confirmed through a self-reported physical activity questionnaire (21, 22) based on the World Health Organization's Physical activity guidelines (at least 150 min of moderate to vigorous activity per week) (23). All the participants received information sheets and signed consent forms as requested by ethical procedures. This study has been reviewed and approved by our local ethical authorities (Local Human Protection Committee - CPP SUD EST VI / CNIL).

### On-site physical activity intervention

Active and inactive groups were requested to take part in two to three training sessions per week, within their worksite training program. Each session lasted 45 min minimum, alternating between muscle-strengthening and cardiorespiratory exercises (one of each per week), supervised by a professional for a duration of 5 months. The program proposed 18 different physical activities such as muscle strengthening, stretching, cardiorespiratory or team sports. The participants were asked to perform at least one muscle-strengthening (weights machines) and one cardiorespiratory session (Latin dance, step, bike, fight exercise…) per week. Compliance was controlled by a computerized access to the sport facilities.

### Anthropometric measurements and body composition

A digital scale was used to measure body mass to the nearest 0.1 kg, and barefoot standing height was assessed to the nearest 0.1 cm using a wall-mounted stadiometer. Both body mass and height were obtained at the same time of the day for the same subject, and not in a fasting state. Body Mass Index (BMI) was calculated as body mass (kg) divided by height squared (m^2^). Body Composition was assessed by bioelectrical impedance analysis, performed with the Tanita MC780 multi frequency segmental body composition analyzer. This analyzer consists in a stand-alone unit where the subject has to step on bare foot (standard mode). Information concerning the subject (age, gender, and height) is entered by the researcher. Once body mass has been assessed by the scale, the subject has to take grips with both hands (alongside his body) during the impedance measure (Hand to foot BIA). A full segmental analysis is performed in less than 20 s. Total body fat, total fat-free mass and body water were reported by the researcher into an excel sheet for statistical treatment. The newly developed BIA analyzer has been recently validated in healthy adults (24).

### Well-being, quality of life and health perception

Well-being and quality of life at work were assessed using a newly developed questionnaire (“worksite well-being and quality of life questionnaire”) especially designed for occupational health studies. The participants were asked to rate statements describing their well-being at work using visual analog scales ranging from ”not at all” to “absolutely” (i.e., “I'm actually feeling distressed while at work”). This questionnaire has been recently validated in a similar population (25).

All participants were also asked to complete a short self-administered questionnaire specifically designed to explore their perception of health (“health perception scale”). Six criteria were investigated: (1) perceived physical fitness, (2) perceived ideal weight, (3) perceived healthy balanced diet, (4) perceived sleep quality, (5) perceived stress level, and (6) perceived general health. A10-point scale from 1 (not at all) to 10 (very much) was used to assess each item. The six individual scores were computed to obtain a global score for health perception. This questionnaire has been previously validated in adults (26).

### Aerobic fitness

The step test was performed on a stool of 16.25 inches (41.3 cm) for men and 11.8 inches (30 cm) for women for a total duration of 6 min at the rate of 24 cycles per minute, which was set by a metronome. Participants were asked to wear a heart rate monitor (Polar Electro Inc, Lake Success; USA) during the test and heart rate was recorded at the end of the 6 min, 30 s and 1 min after completion of the test. This test has been found reliable in healthy subjects and it is highly reproducible (27).

### Muscle capacities

Upper limbs muscle strength was assessed using the handgrip on the dominant hand, as a non-invasive marker of muscle strength of upper limbs, well suitable for clinical use (28). The participants were also asked to perform a maximal number of push-ups (with the knees on the floor) respecting an imposed frequency. The test stopped once they were not able to maintain this rhythmicity or showed difficulties to maintain the correct position (29, 30). Counter Movement Jump (CMJ) was used to assess lower limb muscle strength using the Optojump technology (Microgate SRL, Rome, Italy) (31). The Shirado test was used to assess the static endurance of the abdominal muscles (32) and the Sorensen test to assess back strength and endurance for all the muscles involved in the extension of the trunk (33).

## Statistical considerations

Sample size has been estimated in order to compare overall health indicators as fat mass percentage among physically active and inactive tertiary employees showing a high level of sedentariness. According to our previous work (20) and to Cohen's recommendations (34) who has defined effect-size bounds as: small (ES: 0.2), medium (ES: 0.5) and large (ES: 0.8, “grossly perceptible and therefore large”), we calculated that a minimum of 185 participants would allow to highlight an effect size equal to 0.5 for a two-tailed type I error at 5%, a statistical power of 90% and a ratio 60–40% for inactive/active employees. All analyses were performed using Stata software (version 13, StataCorp, College Station, TX). Statistical analyses were done for a two-sided type I error of a = 5%. Baseline subject's characteristics were presented as the mean ± standard deviation (*SD*) or the [median interquartile range] for continuous data (assumption of normality assessed by using the Shapiro-Wilk test) and as the number of patients and associated percentages for categorical parameters. Quantitative variables were compared (at each time-point evaluation T0 and T1) between independent groups (active vs. inactive tertiary employees) by Student *t*-test or Mann-Whitney test if conditions of *t*-test were not respected (normality and homoscedasticity analyzed using Fisher-Snedecor test). Comparisons between independent groups were done by Chi-squared or when appropriate by Fischer-exact test for categorical variables. To analyze repeated correlated data, the evolution of variations between the beginning and the end of the study was calculated for each parameter. Then, random-effects models were performed to study fixed effects as group (noted intergroup evolution of variations in Tables 1, 2), time-points evaluation and their interaction taking into account between and within subject variability. The normality of residuals was checked for all models. A sensibility analysis was performed to measure the possible impact of missing data (notably imputation of missing data). Results and practical conclusions were analogous (data not shown).

**Table 1:** Intra- and inter-group results for anthropometry and body composition.

		**Inactive**	**Active**	***I vs. A***
Fat mass (%)	T0	24.8 ± 7.5	21.7 ± 7.1	*p = 0.0032*
	T1	22.8 ± 6.9	20.8 ± 7.3	*p = 0.0722*
	*T0 vs. T1*	*p < 0.001*	*p < 0.001*	
Fat-free mass (kg)	T0	52.5 ± 11.3	53.6 ± 10.7	*ns*
	T1	54.5 ± 10.9	52.7 ± 9.9	*ns*
	*T0 vs. T1*	*ns*	*ns*	
BMI (kg/m^2^)	T0	25 ± 4.6	24.1 ± 3.2	*ns*
	T1	24.9 ± 3.9	23.7 ± 3.1	*p = 0.0864*
	*T0 vs. T1*	*ns*	*ns*	
WC (cm)	T0	92 ± 15.3	87.5 ± 12.3	*p = 0.0264*
	T1	92 ± 13.4	87 ± 12.1	*p = 0.0287*
	*T0 vs. T1*	*ns*	*ns*	

*BMI, Body Mass Index; WC, Waist Circumference; T0, baseline; T1, end of the 5-month program; I, Inactive; A, Active*.

**Table 2:** Intra- and inter-group results for functional tests, health perception and well-being.

		**Inactive**	**Active**	***I vs. A***
Health perception	T0	51.2 ± 15.1	61.3 ± 14.3	*p < 0.001*
	T1	59.1 ± 14.7	62.5 ± 12.7	*ns*
	*T0 vs. T1*	*p < 0.001*	*ns*	
Worksite well-being	T0	7.34 ± 1.4	7.3 ± 1.3	*ns*
	T1	7.3 ± 1.2	7.4 ± 1.3	*ns*
	*T0 vs. T1*	*ns*	*ns*	
Rest heart rate (bpm)	T0	71.3 ± 11	67.2 ± 11.6	*ns*
	T1	73.5 ± 10.5	68.3 ± 10.3	*ns*
	*T0 vs. T1*	*ns*	*ns*	
Heart rate (bpm)	T0	150.4 ± 20.2	147.9 ± 19.9	*ns*
	T1	141.1 ± 18.3	135.6 ± 17.3	*p = 0.0698*
	*T0 vs. T1*	*p = 0.0029*	*p < 0.001*	
Heart rate +30 (bpm)	T0	126 ± 26.6	128.3 ± 22	*ns*
	T1	127.8 ± 19.3	116.9 ± 19.2	*p = 0.0329*
	*T0 vs. T1*	*ns*	*p < 0.001*	
Heart rate +60 (bpm)	T0	116 ± 20.8	112.4 ± 22.4	*ns*
	T1	111.3 ± 21.3	103 ± 18.4	*p = 0.0236*
	*T0 vs. T1*	*ns*	*p < 0.001*	
CMJ (cm)	T0	24.6 ± 7.7	23.6 ± 7.1	*ns*
	T1	26.7 ± 7.6	24.4 ± 8.9	*ns*
	*T0 vs. T1*	*p = 0.0256*	*ns*	
Handgrip (kg)	T0	24.6 ± 7.7	23.6 ± 7.1	*ns*
	T1	41.8 ± 10.7	40.3 ± 11.1	*ns*
	*T0 vs. T1*	*p = 0.0035*	*p = 0.0082*	
Puch-ups (rep)	T0	22.8 ± 14.4	36.5 ± 19.2	*p < 0.001*
	T1	29.4 ± 15.4	39.8 ± 18	*p = 0.0014*
	*T0 vs. T1*	*p < 0.001*	*ns*	
Shirado (s)	T0	173.1 ± 77.7	181.8 ± 50.9	*ns*
	T1	184.1 ± 82.2	174.3 ± 52	*ns*
	*T0 vs. T1*	*ns*	*ns*	
Sorensen (s)	T0	101.2 ± 56.9	159 ± 41.6	*p < 0.001*
	T1	112.3 ± 54.2	161.2 ± 47.7	*p < 0.001*
	*T0 vs. T1*	*ns*	*ns*	

*CMJ, Counter Movement Jump; ST, step test; HR, heart rate; T0, baseline; T1, end of the 5-month program; I, Inactive; A, Active*.

## Results

A total of 193 middle and upper classes office workers took part in the study; 84 females and 110 males with an average age of 44.2 ± 9.8 years, an average weight and BMI of 72.6 ± 14.7 kg and 24.5 ± 3.8 kg/m^2^ respectively. At baseline (T0), 98 and 95 participants composed the inactive and active groups respectively against 71 and 73 at the end of the intervention (T1).

**Figure 1:**
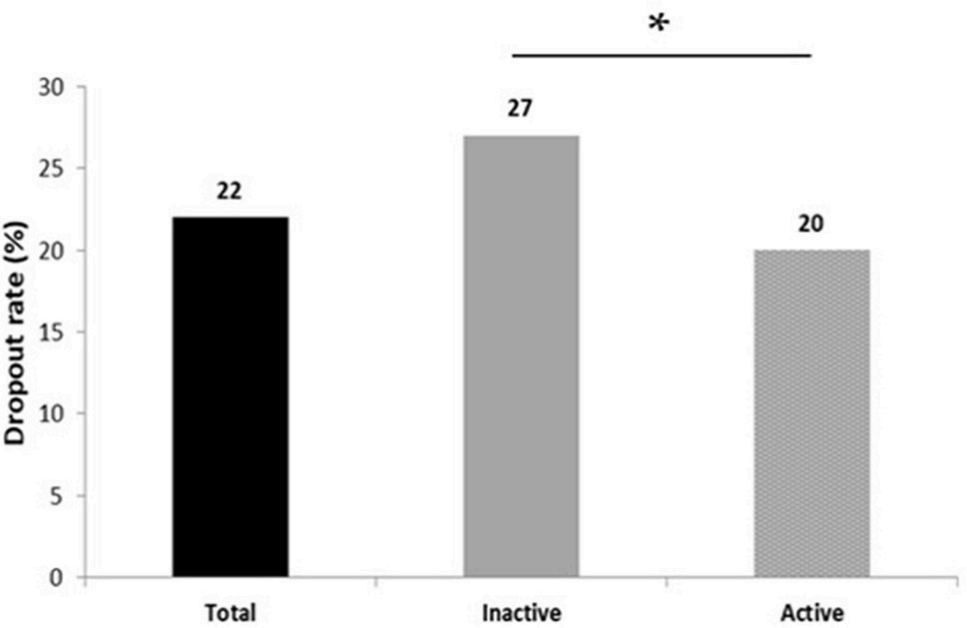
Dropout rates to a 5-month workplace physical activity program between initially active and inactive tertiary employees (*p ≤ 0.05).

Figure 1 illustrates the dropout rate observed during the 5-month workplace program. 22% of all subjects dropped out before the end of the intervention, with a higher rate observed in the initially active employees: 20% did not complete the whole study against 27% in the inactive sample (*p* < 0.05).

Table 1 details the results for anthropometric characteristics and body composition, while Table 2 presents the participants' functional characteristics, well-being and health perception. There was a statistically significant difference for push-ups (*p* ≤ 0.001; *p* ≤ 0.01 respectively), Sorensen test (*p* ≤ 0.001), fat mass (*p* ≤ 0.01; tendency respectively) and waist circumference (*p* ≤ 0.05) between active and inactive at T0 but also at T1. Table 2 also highlights a significant difference for health perception at T0 between active and inactive participants (*p* ≤ 0.001), but this difference no longer exists at T1. No significant change in worksite well-being, rest heart rate, CMJ, Shirado test, fat-free mass or BMI was observed during the study. There was a time effect for the inactive group which demonstrates significant progressions between T0 and T1 for health perception (*p* ≤ 0.001), and CMJ (*p* ≤ 0.01). In contrast, the two groups significantly improved their results for handgrip (*p* ≤ 0.01), push-ups (inactive: *p* ≤ 0.001 and active: *p* ≤ 0.05), fat mass (*p* ≤ 0.001) and their heart rate post exercise (inactive: *p* ≤ 0.01 and active: *p* ≤ 0.001) between T0 and T1.

Table 3 details the results of the amplitude of the variations obtained between T0 and T1. Health perception (*p* ≤ 0.001), performances at the Sorensen test (*p* ≤ 0.05), Shirado test (tendency) and BMI (tendency) all show a significantly higher progression in the inactive compared with the active groups. The variations observed between T0 and T1 were not found significantly different between groups for worksite well-being, CMJ, handgrip, push-ups, fat mass, fat-free mass and waist circumference.

**Table 3:** Intergroup evolution of variations between the beginning and the end of the study.

	**Inactive**		**Active**		***p***
	**mean sd**	**var**	**mean sd**	**var**	
Health perception	20.2 ± 35.4	13.6 [−2.7;35.1]	3.7 ± 21.2	1.5 [−11;8.4]	*p* < 0.001
Worksite well-being	2 ± 21.3	1.4 [−11.4;14.3]	1.8 ± 17.2	−0.06 [−6.4;8.3]	
CMJ (cm)	5.2 ± 14	3.2 [−3.8;9.9]	3.9 ± 21.8	3.3 [−1.6;12.2]	
Handgrip (kg)	3.4 ± 8.7	2.9 [−1.5;9.1]	4.1 ± 10.9	3.2 [−3.1;10.3]	
Puch-ups (rep)	29.5 ± 65.3	16.4 [0;40]	50 ± 230.2	6 [−5.9;26]	
Shirado (sec)	20.5 ± 73	0 [−14.3;28.7]	−2.9 ± 22.4	0 [0;0]	*p* = 0.0743
Sorensen (sec)	35.4 ± 119.8	0 [0;28.2]	0.08 ± 21.7	0 [0;0]	*p* = 0.0341
Fat mass (%)	−4.2 ± 9.3	−4.1 [−9.6;−0.4]	−3.4 ± 8.9	−2.7 [−7.8;3.3]	
Fat-free mass (kg)	0.8 ± 2.6	1 [−0.4;2.4]	0.7 ± 3.4	0.7 [−0.5;2.4]	
BMI (kg/m^2^)	−0.6 ± 3	−0.4 [−2;0.8]	0.1 ± 2.4	0.4 [−1.3;1.7]	*p* = 0.0612
WC (cm)	−0.2 ± 3.6	-0.4 [−2.3;1.5]	0.05 ± 3.6	−0.1 [−2.5;2.8]	

*BMI, Body Mass Index; WC, Waist Circumference; CMJ, Counter Movement Jump; T0, baseline; T1, end of the 5-month program; I, Inactive; A, Active*.

## Discussion

The main aim of the present study was to compare overall health indicators between active (above recommended levels of physical activity) and inactive (below recommended levels) employees with high and similar levels of worksite-induced sedentariness. According to our results, BMI, fat-free mass, lower and upper limbs muscle capacities (CMJ and handgrip) and aerobic capacities are not different between active and inactive tertiary employees who both spend about 7.5 h per days seated in front of computers (approximately 37.5 h/week). This is to our knowledge the first study that questions the effect of a high level of sedentariness on health and fitness indicators among physically active and inactive workers.

While public health strategies have been developed to increase individuals' physical activity levels over the last decades, more concerns are expressed today regarding the progression of sedentariness. Indeed, it is now clear that physical activity and sedentary behaviors are two different constructs that have independent effects on health (35). This is particularly important since sedentariness has become one of the largest public health concerns, being nowadays recognized as one of the main causes of preventable premature mortality. Although worksites have been identified as ideal settings to favor physical activity among workers (9), the “tertiarisation” of our societies and industries has been favoring sedentariness, with employees spending now most of their daily time seated in front of computers. While most of the available studies have been evaluating the levels of physical activity of tertiary employees (36), their time spent seated and/or in front of a computer screen (37), or have been independently looking at the effect of physical activity programs (38) or interventions aiming at reducing sedentary time (39), we did not find any work comparing fitness parameters between physically active and inactive tertiary employees who are characterized by a high level of sedentariness.

In their study, Ko et al. compared the incidence of metabolic risk factors and the prevalence of metabolic syndrome between white collars divided in physical activity tertiles (40). According to their results, workers with the lowest physical activity levels present higher waist circumference, increased triglycerides concentrations, higher HDL-C concentrations and a higher risk for metabolic syndrome (40). Unfortunately, their data do not allow the classification of their participants as active or inactive in regards to physical activity guidelines, and information regarding sedentary behaviors is missing, as well as details regarding the exact occupational activity of their participants (40). More recently, Browne et al. conducted a cross-sectional study among about 500 sedentary tertiary employees, hypothesizing that sedentary occupational workers who meet physical activity recommendations (without precising the guidelines used) present lower risks for metabolic syndrome than inactive ones (41). Their results point out that active workers show lower odds for abdominal obesity, elevated blood pressure, reduced high-density lipoprotein cholesterol and overall metabolic syndrome, after adjustments for age, working hours, body mass index, and tobacco use (41). The significantly higher fat mass percentage and waist circumference observed in our inactive group are in line with these results. Although these studies tend to suggest that in employees who spend most of their daily time sedentary, physical activity has beneficial effect on their metabolic health, we missed finding any data related to physical and overall health fitness.

Not only objective health and fitness indicators were assessed in the present work, employees' self-perception of their overall health and work-related well-being were investigated. Although active workers show a significantly greater score for overall health perception, worksite wellbeing was not significantly different compared with inactive employees. Although this might suggest that physical activity levels might not be associated with employees' work-related satisfaction and comfort, some recent studies suggest that tertiary employees' wellbeing might be related to sedentary behaviors, which may explain our results since both active and inactive workers present here at least 7.5 h of sedentary time per day (42). Such results are definitely of importance for both workers and employers, since work-related wellbeing has been clearly identified as a major predictive factor for prolonged or future sickness absence (43).

The second aim of the present work was to assess the effects of a 5-month worksite structured and monitored physical activity program among initially inactive and active employees (already regularly using the companies' physical facilities for the last 2 years). While the majority of the published papers in the field assesses the effects of physical educations, encouragement and motivational strategies (39, 44) walking meetings (45) or sedentary breaks (46), less studies have been conducted implementing structured aerobic and resistance exercise sessions (20, 47). In 2009, Pedersen et al. conducted a 1 year randomized controlled trial questioning the effects of resistance training and all-round physical exercise sessions performed within workplaces (1 h/ week during working hours) on tertiary employees health indicators (47). Their results showed significant reductions of cardiovascular and metabolic syndrome-related risk factors as well as musculoskeletal pain symptoms, concomitantly with minor increases in physical capacities. The physical activity levels of the participants was however not considered. In the present study, the participants were asked to perform 2 to 3 exercise sessions per week, composed of resistance and aerobic exercises, in one of the physical activity facilities proposed by their companies. According to our results, while numbers of health and fitness indicators were improved by the program in our initially inactive subsample, active individuals also show improvements. While health perception and counter movement jumps were significantly improved in the inactive group only, the heart rate response to our aerobic test, the handgrip performance, the maximal number of push-ups, as well as the percentage of fat mass was significantly improved in both groups. Interestingly, the heart rate recovery 30 s and 1 min after the aerobic test were improved in the experienced group only. These results suggest that while such a physical activity intervention has beneficial effects in initially inactive employees, it continues to favor positive adaptations in active ones. These results are in line with a previously published pilot study, suffering from reduced sample size but already suggesting the interest of worksite exercise programs among tertiary employees, whatever their initial physical activity level (20).

Although the present study provides main insights regarding the importance of considering both sedentary and physical activity levels when it comes to evaluating tertiary employees' health indicators, and underlines the beneficial effects of structured on-site physical interventions among both active and inactive workers, our results also point out the necessity to consider the employees' adherence rate to such programs. Indeed, 1 out of 5 participants did not complete the whole intervention, which must definitely be considered by stakeholders and investigators who must try to understand and identify the potential undelying reasons. This 20% dropout rate observed here, is in line with what is rarely discussed but usually observed in other similar studies. In their study, Jakobsen et al. asked tertiary employees to exercise 5 times a week (10-min sessions) for 10 weeks and observed a similar dropout rate of 22% (48). During their 1 year trial, Pedersen et al. obtained a 48% dropout rate among office workers who exercised 3 times a week (47) and in our previous pilot work, 30% of the enrolled participants were found to quit before the end of the intervention (20). While such high dropout rates are usually observed, the profile of these participants remains under-explored. Although further studies are needed, specifically designed to address this question, our results, as illustrated by the Figure 1, tend to suggest that the initial physical activity level of the enrolled employees should be considered with a total of 20% of non-compliant being observed in the initially active sub-sample against 27% among the initially inactive one. This also highlights the fact the least active employees–and therefore those who need it the most—are those who are at highest risk to drop out from such worksite PA programs.

Our results should clearly be interpreted in light of some limitations. The sample size might be considered as one of the limitations, underlying the difficulty to recruit volunteers for such interventions, reinforcing the need for deeper explorations of the potential specific profile of employees interested in workplace physical activity programs. The use of field testing to assess employees' physical fitness might also be a limitation, and more objective methods could be used, such as ergometers and direct methods to assess their aerobic capacities (laboratory-based maximal aerobic testing). Moreover, the use of BIA to assess body composition is not as accurate as dual-x-ray absorptiometry for instance; it composes however one of the best alternatives for studies enrolling large samples and remains a reliable and validated tool in healthy adults as previously described (24).

To conclude, the present study suggests that improvements in some health and fitness indicators might not be found among active tertiary employees, compared with inactive ones, which might be due to the high level of sedentariness characterizing their occupational task. This result clearly calls for worksite-based interventions not only focusing on physical activity but also, and perhaps most importantly, trying to break down sedentary time. Our results also confirm that structured exercise interventions implemented with workplaces, improve health and fitness among both initially physically inactive and active tertiary workers, questioning however the profile of workers who are willing to be compliant to on-site physical interventions.

## Author contributions

PG and BP analyzed the data used in the manuscript, PD and JF participated in the project design. PG, DT, FD, and MD wrote and reviewed the paper.

### Conflict of interest statement

The authors declare that the research was conducted in the absence of any commercial or financial relationships that could be construed as a potential conflict of interest.
